# Emergence of the invasive Asian bush mosquito, *Aedes* (*Finlaya*) *japonicus japonicus*, in an urban area, Romania

**DOI:** 10.1186/s13071-021-04698-2

**Published:** 2021-04-07

**Authors:** Cintia Horváth, Cristina Daniela Cazan, Andrei Daniel Mihalca

**Affiliations:** 1 Department of Parasitology and Parasitic Diseases, University of Agricultural Sciences and Veterinary Medicine of Cluj-Napoca, Calea Mănăştur 3-5, 400372 Cluj-Napoca, Romania; 2 CDS-9, “Regele Mihai I al României” Life Science Institute, University of Agricultural Sciences and Veterinary Medicine of Cluj-Napoca, Calea Mănăștur 3-5, 400372 Cluj-Napoca, Romania

**Keywords:** *Aedes japonicus*, Airport, Asian bush mosquito, Invasive species, Surveillance

## Abstract

**Background:**

A study conducted at the International Airport of Cluj-Napoca, Romania, with the aim of investigating the presence/absence of invasive *Aedes* mosquito species resulted in finding *Aedes japonicus japonicus* (Theobald 1901) eggs in one of the ovitraps placed on site.

**Methods:**

The study was carried out between 30 June and 29 September 2020. On 24 August, 26 eggs were collected and later hatched at the University of Agricultural Sciences and Veterinary Medicine of Cluj-Napoca’s insectary. On 15 October another adult female *Ae. japonicus* was caught entering a building in the center of the city, about 7 km from the first sampling spot.

**Results:**

The mosquitoes were identified morphologically and confirmed by molecular analysis, based on the genetic analysis of the mitochondrial gene cytochrome *c* oxidase subunit 1 (COI).

**Conclusion:**

This is the first report of the species in Romania, highlighting the need for surveillance and implemented control methods. However, in Romania to our knowledge only *Aedes albopictus* has been established; further studies are required to learn about this new invasive species' status in Romania.

**Graphical Abstract:**

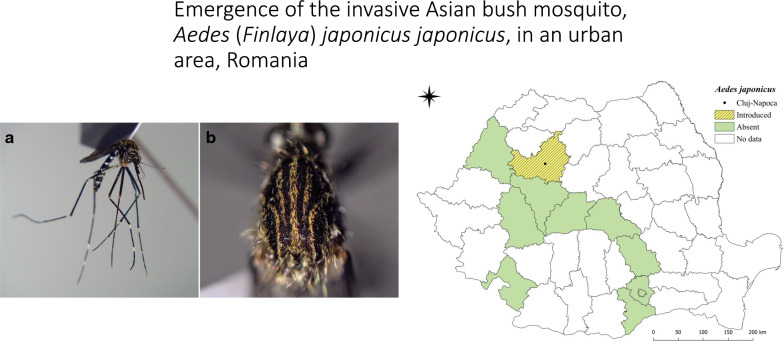

## Background

The Asian bush mosquito, *Aedes* (*Finlaya*) *japonicus japonicus* (Theobald 1901) (Diptera: Culicidae), is naturally occurring in Southeast Asia (Palaearctic Japan, Korea, parts of China, Taiwan and Russia) [1]. In the past 3 decades, it has become invasive in various parts of the world, including Central Europe. Its invasion was mostly mediated by anthropogenic movements. Like for the related species, *Aedes albopictus*, it has travelled throughout the continents mainly via the trade of used tires, which is considered the most important introduction means of these container breeding species [2, 3].

The first report of *Ae. japonicus* occurring outside its endemic area came from New Zealand [4], where during an entomological survey, in 1993, the species was found in a shipment of used tires together with *Ae. albopictus*. Later on, in 1998, it was reported in the USA (New York and New Jersey) [5] and Canada [6]. The first record of the species in Europe dates back to 2000, when two larvae were collected in used tires in a storage yard from a metropolitan area of France [3]. Later, the species was eradicated, just to return and establish a decade later [7]. *Ae. japonicus* is showing rapid expansion throughout Europe, and in less than 2 decades it has managed to become established in 13 countries (Table 1). Its success lays in the species' ecological flexibility, its ability to adapt to new climatic conditions and larval habitats, and its capacity to overwinter [8]. Its tolerance to lower temperatures is responsible for a wider distribution range compared to other invasive species, indicating a bigger colonizing potential in the future [9].

**Table 1:** Status of *Ae. japonicus* in Europe

Year of first observation	Country	Current status	References
2000	France	Established	Schaffner et al. [3]
2002	Belgium	Established	Versteirt et al. [13]
2008	Switzerland	Established	Schaffner et al. [14]
2008	Germany	Established	Schaffner et al. [14]
2011	Austria	Established	Seidel et al. [15]
2011	Slovenia	Established	Seidel et al. [15]
2012	The Netherlands	Established	Ibáñez-Justicia et al. [16]
2012	Hungary	Established	Seidel et al. [17]
2013	Croatia	Established	Klobučar et al. [18]
2015	Liechtenstein	Established	Seidel et al. [17]
2015	Italy	Established	Montarsi et al. [19]
2017	Bosnia and Herzegovina	Introduced	Janssen et al. [20]
2018	Serbia	Introduced	Janssen et al. [20]
2018	Spain	Established	Eritja et al. [21]
2018	Luxembourg	Established	Schaffner and Ries [22]
2020	Romania	Introduced	Current study

Its invasion is supported by globalization; the excessive trade of used tires and increasing tourism both play a considerable role in the colonization process [10]. However, *Ae. japonicus* seems to prefer rural habitats over urban sites [11]. It uses different types of natural and artificial larval habitats: tree-holes, rock pools, ponds, ditches, rain water pools, tires, cans, bird baths, water barrels, buckets, etc. The larvae usually feed on decaying organic matter and algae [8]. It is often found together and competes for the breeding sites with other container-breeding mosquitoes [12]. Therefore, *Ae. japonicus* can be a challenge for local mosquito populations. Although *Ae. japonicus* is not considered a major disease vector, it is known to transmit several flaviviruses (i.e. West Nile, Japanese encephalitis, chikungunya, dengue, LaCrosse, Eastern equine encephalitis, St. Louis encephalitis, Rift Valley fever, Zika and Usutu viruses) and nematodes such as *Dirofilaria repens* and *D. immitis* [23–33]. According to the ECDC’s Annual Epidemiological Report for 2018 [34], Romania has a significant number of cases of West Nile encephalitis, with the highest numbers in 2018 (267 confirmed cases with 43 deaths). Recently, imported cases of dengue [35], Zika [36], yellow fever [37] and chikungunya [38] were reported in Romania. With the generally increased tourism (with the exception of 2020 COVID-19-related travel bans and restrictions), the emergence of exotic diseases is an ever-present risk. This, in connection to the spread of invasive mosquitoes [39], is causing a permanent epidemiological concern. The airports and harbors seem to be particularly sensitive regions for the introduction and establishment of exotic mosquito species like *Ae. japonicus*; therefore, they need careful observation [40, 41]. For this reason, we conducted a targeted surveillance study to detect adult and immature stages of invasive mosquitoes at an international airport in Romania as part of harmonized mosquito monitoring (www.aedescost.eu/aimsurv). This article is the first report of *Ae. japonicus* in Romania.

## Methods

### Study area and design

**Fig. 1:**
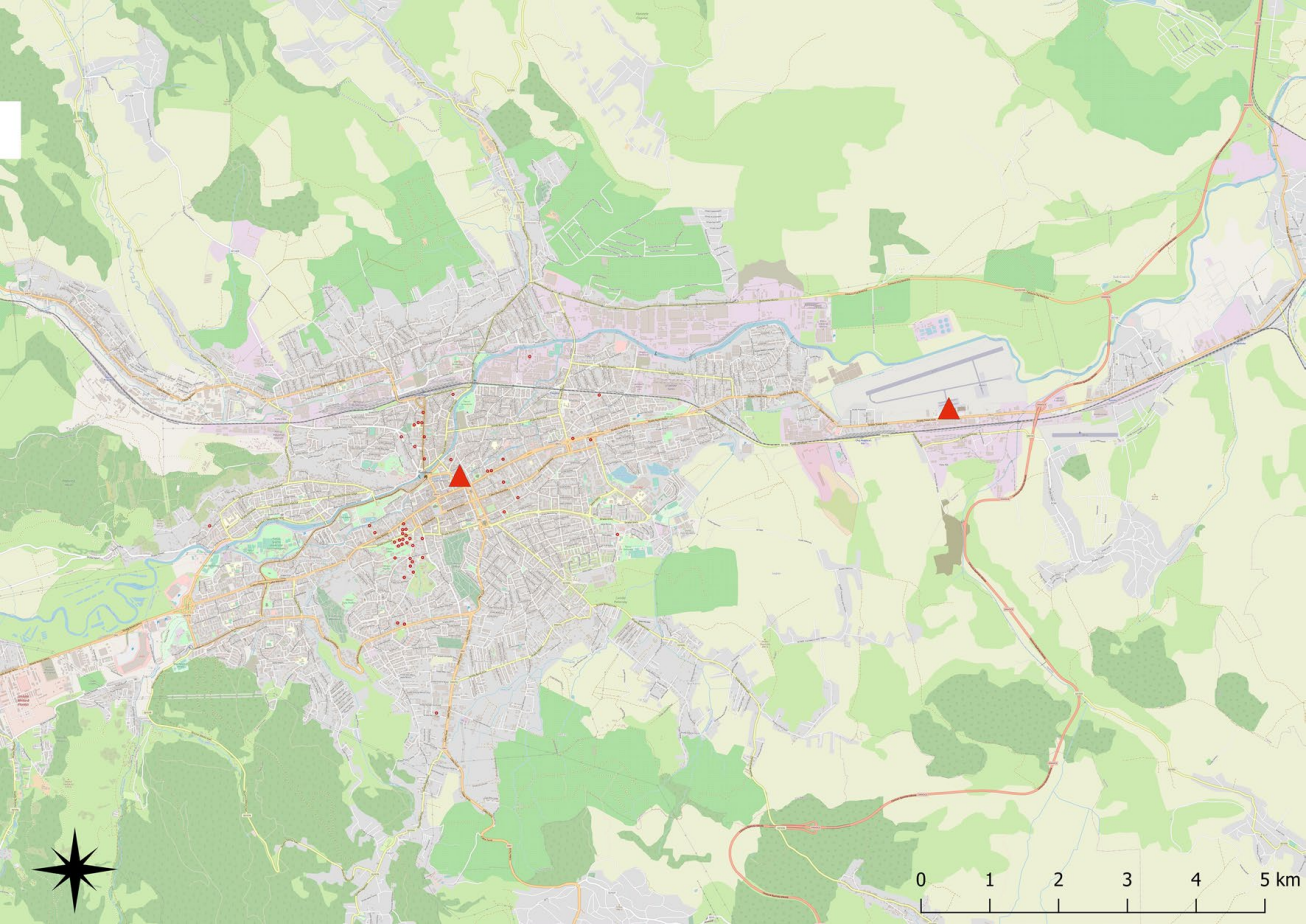
Location of the study area in Cluj-Napoca, Romania. Red triangles indicate the sampling locations

An entomological survey was conducted for 3 months (between 30 June and 29 September 2020) at the ‘Avram Iancu’ International Airport in Cluj-Napoca, Romania, to detect invasive *Aedes* species (Fig. 1). The study was part of a coordinated Pan-European initiative within the framework of Aedes Invasive Mosquitoes COST Action (www.aedescost.eu/aimsurv). One BG-Sentinel trap baited with BG-Lure (Biogents AG, Regensburg, Germany) and dry ice was set at the airport. The dry ice was placed in a special storage unit inside the trap, which allowed it to sublimate gradually, releasing gaseous carbon dioxide (CO_2_) while the trap was operating. Additionally, five ovitraps were also set up at the investigated area and positioned approximately 50 m apart to increase the sensitivity of detection. The ovitraps were represented by 1-l black plastic recipients filled with tap water (approximately 700 ml) and a piece of polystyrene (ca. 10 cm × 5 cm × 2 cm) as an oviposition substrate.

The traps were set in the airport apron areas where aircraft were parked while on land and by selecting areas where vegetation was present. Each week, the BG-Sentinel trap was operating for 1 night (approximately 15 h), usually from Monday evening (7 p.m.) until Tuesday morning (10 a.m.) when mosquitoes were collected. Ovitraps were permanently in place, and the oviposition substrate was checked for the presence of eggs and replaced every second week. The eggs were later hatched at the University of Agricultural Sciences and Veterinary Medicine of Cluj-Napoca’s insectary by submerging the polystyrene pieces in tap water in a controlled environment, where the room temperature was set at 30 °C and the humidity at 55%. Larvae were fed with TetraMin Mini Granules (Tetra GmbH, Germany). Moreover, another female mosquito resembling an invasive species was accidentally caught inside a building in Cluj-Napoca’s central area, spontaneously and unrelated to the study design.

### Morphological identification

Adult mosquitoes were collected and identified using the morphological key by Becker et al. [42]. The adult mosquitoes which emerged from eggs were identified using the key in the European Centre for Disease Prevention and Control’s guidelines for the surveillance of the invasive mosquitoes in Europe (2012) [43]. The mosquitoes were cooled to 4 °C in a refrigerator and identified.

### Molecular assessment

For the adult mosquitoes for which the morphological examination to the species level was impossible because of incomplete body parts, a molecular identification protocol was applied. The DNA was extracted individually from each female mosquito specimen using ISOLATE II Genomic DNA Kit (Bioline Meridian Bioscience, Luckenwalde Germany), according to the manufacturer’s instructions and stored at − 20 °C.

PCR amplification of the cytochrome c oxidase subunit 1 (COI) gene region (~ 660 bp) was performed in 25 µl reaction volume, containing 12.5 µl Red PCR Mastermix (Rovalab GmBH, Teltow, Germany), 6.5 µl of ultra-pure water, 1 µl (10 pmol/μL) of each of the two primers LCO1490 (5′-GGTCAACAAATCATAAAGATATTGG-3′) and HCO2198 (5′-TAAACTTCAGGGTGACCAAAAAATCA-3′) [44] and 4 µl aliquot of isolated DNA. One negative control (ultra-pure water) was included.

The PCR was performed using the T1000™ Thermal Cycler (Bio-Rad, London, UK) with the following conditions: initial denaturation at 95 °C for 5 min, followed by 40 cycles of denaturation at 95 °C for 45 s, annealing at 47 °C for 45 s and extension at 72 °C for 45 s, with a final extension at 72 °C for 5 min. Amplification products were visualized by electrophoresis on 1.5% agarose gel stained with ECO Safe 20,000× Nucleic Acid Staining Solution (Pacific Image Electronics, New Taipei, Taiwan), and their molecular weight was assessed by comparison to a molecular marker (O’GeneRuler™ 100 bp DNA Ladder, Thermo Fisher Scientific Inc., Waltham, MA, USA). PCR products were purified using the ISOLATE II PCR and Gel Kit (Bioline Meridian Bioscience, Luckenwalde Germany) and sent for sequencing (Macrogen Europe, Amsterdam, The Netherlands).

The sequences were compared with those available in GenBank™ using Basic Local Alignments Tool (BLAST) analyses. All sequences were analyzed and edited using Geneious^®^ 4.85 software [45].

### Mapping

The maps were generated using QGis 3.6.2 software (http://www.qgis.org).

## Results

**Fig. 2:**
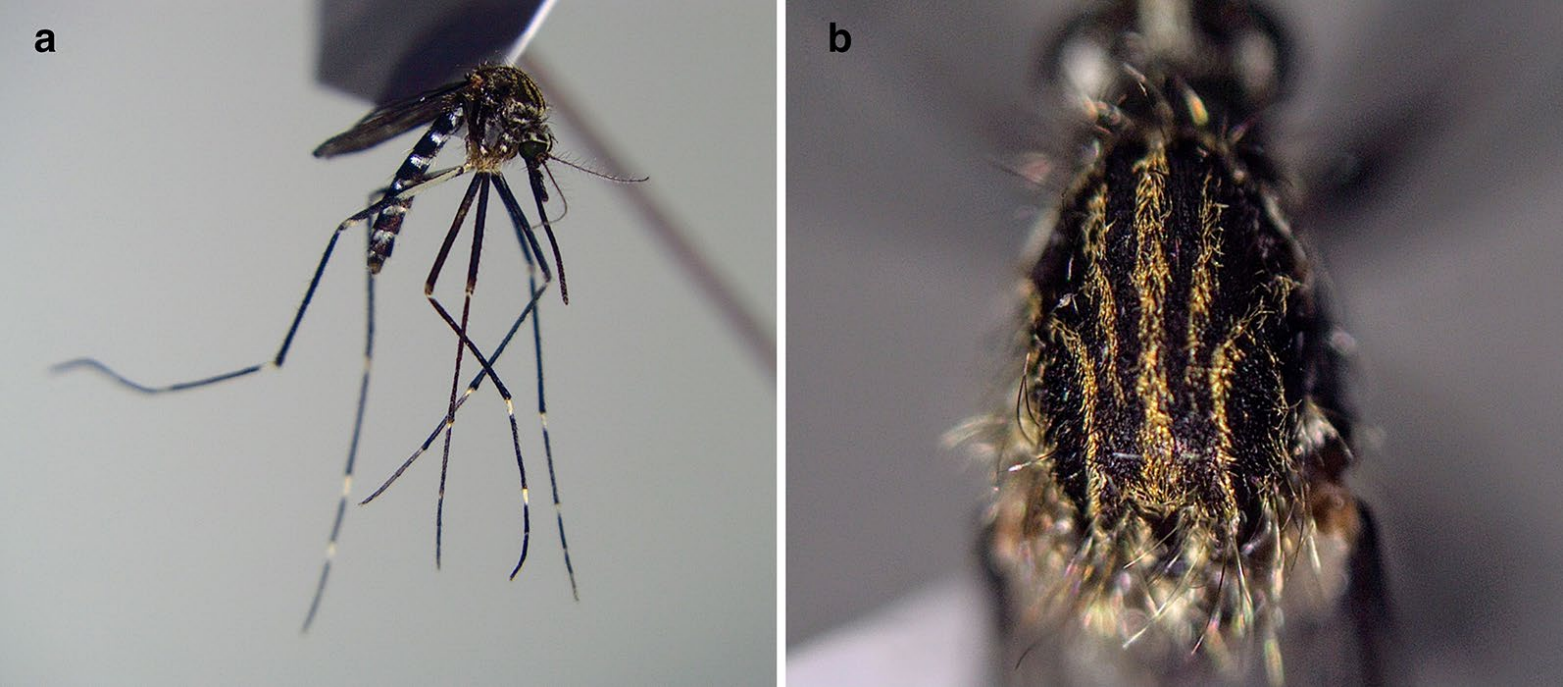
Specimen of *Aedes japonicus* collected at the airport in 2020: **a** lateral view; **b** scutum

Mosquito eggs were found only on 24 August 2020 when 26 eggs were collected from one of the five traps (46.782228° N, 23.686877° E). The rearing procedure resulted in seven adult mosquitoes, two males and five females (Fig. 2). The COI sequence analysis showed a similarity of 100% with other *Ae. japonicus* specimens from Germany (accession numbers KF211480.1, KP076236.1, KP076252.1).

No adult *Ae. japonicus* were sampled in the BG-Sentinel trap, but females and males of *Culex pipiens* and *Ochlerotatus caspius* were collected (results not shown).

On 15 October, another female of *Ae. japonicus* was caught accidentally inside a building in Cluj-Napoca’s central area (46.776425° N, 23.593782° E). For this specimen the morphological examination to species level was impossible because of incomplete body parts; therefore, molecular analysis was performed to identify the species. The specimen showed a 100% match with specimens from Austria (accession number MN103386.1), the USA (accession number GQ254794.1), Japan (accession number LC341253.1), Germany (accession number KP076233.1, KP076256.1, KX260924.1, KX260920.1), Canada (accession number MN058628.1) and Italy (accession number MK265686.1, MK265689.1, MK265688.1). All sequences were submitted to the GenBank database under the accession numbers MW509625 and MW509626.

## Discussion

Over the past few decades, five mosquito species have become invasive in Europe [46]. Among these, three species in particular are of concern and seem to be rapidly expanding their range: *Ae. japonicus*, *Ae. albopictus* and *Ae. koreicus*. Such invasive mosquitoes represent a significant ecological and epidemiological threat generating one of the most challenging public health issues nowadays. To date, in Romania only *Ae. albopictus* has been found and is considered established [39, 47]. Mostly because these invasive mosquitoes share similar niches [8], establishment of other invasive species is expected. A climatic model by Cunze et al. [10] predicted that *Ae. japonicus* could also occur at the inner part of the Carpathian Basin under similar conditions as other established populations in Europe. These predictions match with our findings as well, although we cannot yet confirm this species establishment. As *Ae. japonicus* is assumed to be adapted to temperate climatic conditions [8], further surveillance should be continued to establish the species status in Romania.

Compared to other invasive species, *Ae. japonicus* has an advantage because it has a longer activity period over the year, its life-cycle is multivoltine and it overwinters as eggs [8, 48]. Blood meal analysis in wild-caught females showed that they prefer feeding on humans and other mammalian hosts (such as horses, deer, opossums, chipmunks), but also on birds. Females are not considered aggressive biters; however, they readily feed on hosts and they show crepuscular activity [49].

*Aedes japonicus* is part of a species group with four allopatric subspecies, *Ae. j. japonicus*, *Ae. j. shintienensis*, *Ae. j. yaeyamensis* and *Ae. j. amamiensis* [1, 8]. They share similar biology and morphology, but only *Ae. j. japonicus* has become invasive. *Aedes koreicus*, a closely related sibling species invasive in Europe, is naturally endemic to East Asia [1].

**Fig. 3:**
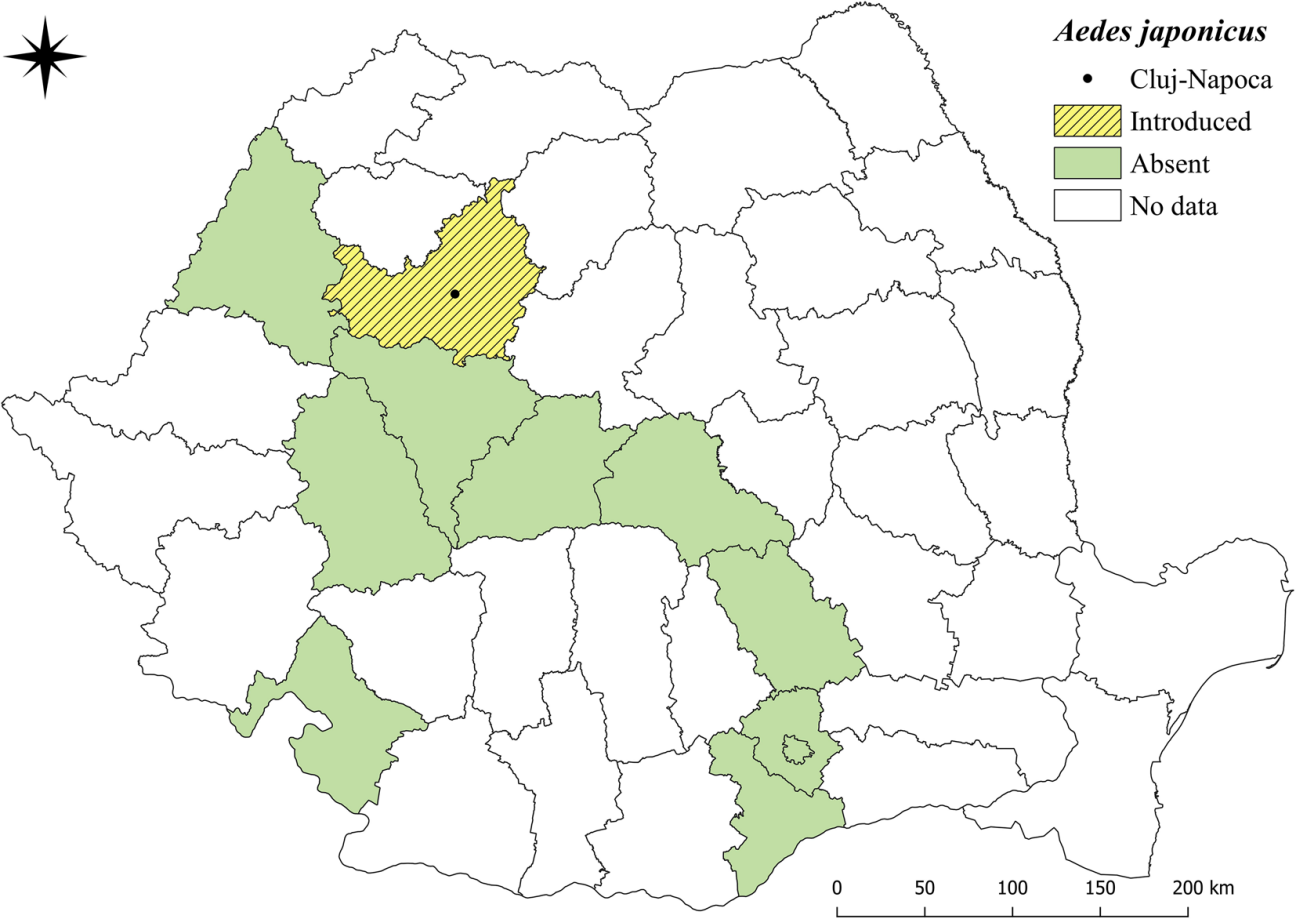
Current known distribution of *Aedes japonicus* in Romania (counties shown in green with confirmed absence according to the European Centre for Disease Prevention and Control and European Food Safety Authority. Mosquito maps [internet]. Stockholm: ECDC; 2020. Available from: https://www.ecdc.europa.eu/en/publications-data/aedes-japonicus-current-known-distribution-may-2020)

The present study reports for the first time the presence of *Ae. japonicus* in Romania (Fig. 3). Moreover, it represents the first invasive species of mosquito detected in Cluj-Napoca, the second most populous city of Romania [50]. Due to the study design, adult and egg trappings were limited to suitable habitats inside the airport. Further studies and long-term surveillance are required for clarifying the introduced or established status of this mosquito species. Due to its climatic and environmental conditions, Romania has a high probability of being invaded by *Ae. japonicus* in the future. Distribution data show that attention should be paid also to the possible presence of another invasive species, *Ae. koreicus*, which is present in Hungary [51]. Like the closely related *Ae. japonicus*, *Ae. koreicus* is also a container-breeding mosquito species that is well adapted to temperate climates. A more accurate picture of the threats that these invasive species pose could be the fact that *Ae. albopictus*, after only 8 years from its first finding in Bucharest [47], has now become established in eight counties in Romania [39].

These new findings highlight the need for control measures to be implemented in the affected areas, and further surveillance should be carried out. A first step in the management of invasive species in Romania could be the implementation of regular entomological surveillance to keep these species under control and educating citizens about the reduction of potential breeding sites and involving them into surveillance programmes.

## Conclusions

We are reporting for the first time to our knowledge the presence of *Ae. japonicus* in Romania. This study was designed to be part of a harmonized Pan-European surveillance coordinated by the AIM-Cost program. Our findings draw attention to the dissemination of invasive mosquito species, which, together with increased global travel and trade, as well as climate change, presents a significant risk to the public health system and economy. Invasive species expand their geographic range steadily, increasing the circulation of vector-borne diseases globally. We recommend that future investigations should be carried out in Romania and elsewhere to assess the distribution of invasive mosquito species and make local authorities aware of the situation so they can carry out regular surveillance and mosquito control in the affected areas.

## Data Availability

The data supporting the conclusions of this article are included within the article.
