# Correction to: Emergence of the invasive Asian bush mosquito, *Aedes* (*Finlaya*) *japonicus japonicus*, in an urban area, Romania

**DOI:** 10.1186/s13071-021-04730-5

**Published:** 2021-04-30

**Authors:** Cintia Horváth, Cristina Daniela Cazan, Andrei Daniel Mihalca

**Affiliations:** 1 Department of Parasitology and Parasitic Diseases, University of Agricultural Sciences and Veterinary Medicine of Cluj-Napoca, Calea Mănăştur 3-5, 400372 Cluj-Napoca, Romania; 2 CDS-9, “Regele Mihai I al României” Life Science Institute, University of Agricultural Sciences and Veterinary Medicine of Cluj-Napoca, Calea Mănăştur 3-5, 400372 Cluj-Napoca, Romania

## Correction to: Parasites Vectors (2021) 14: 192 https://doi.org/10.1186/s13071-021-04698-2

Following publication of the original article [1], it was brought to our that the following sentence had been omitted from the Acknowledgements of the article:

“The work of Cintia Horváth and Andrei Daniel Mihalca was done within the framework of AIM-COST Action CA17108.”

The original article has since been corrected.

The publisher apologizes for any inconvenience caused.
